# Toward culturally responsive psychology higher education courses: psychologists’ perspectives on preparedness to work with Aboriginal and Torres Strait Islander clients

**DOI:** 10.1080/00049530.2025.2474546

**Published:** 2025-03-12

**Authors:** Emily Darnett, Andrew Peters, Monica Thielking

**Affiliations:** a Department of Psychological Sciences, Swinburne University of Technology, Melbourne, Australia; b School of Psychology and Counseling, Queensland University of Technology, Brisbane, Australia; c Indigenous Studies, Swinburne University of Technology, Melbourne, Australia; d School of Psychology and Public Health, La Trobe University, Melbourne, Australia

**Keywords:** Curriculum reform, cultural responsiveness, indigenous psychology, higher education

## Abstract

**Background:**

Psychology course regulatory standards for Australian universities have evolved in that universities are required to include cultural responsiveness in psychology curriculum and demonstrate graduate competencies for working with Aboriginal and Torres Strait Islander clients.

**Aim:**

This study aimed to explore psychologists’ perspectives about the higher education (HE) psychology curriculum in relation to their preparedness to practice with Aboriginal and Torres Strait Islander clients and share their suggestions for improving cultural responsiveness and preparedness.

**Method:**

Psychologists (*N*=108, Female 83.2%, Male 16.8%, Aboriginal 13.9%, non-Indigenous 86.1%, age range 22–83) responded to an electronic mixed-method survey.

**Findings:**

The majority of participants (91.43%, including all Aboriginal psychologists) reported that their psychology HE training did not adequately prepare them to work with Aboriginal and Torres Strait Islander clients. Moreover, 87.5% (Group 3 *n*=16) reported apprehensions about working with Aboriginal and Torres Strait Islander clients for the first time. Limited understanding of culture, concerns for their competence, or worry about perpetuating harm underpinned psychologists’ apprehensions. Most participants (90.5%, *n*=85) indicated they plan to increase their knowledge in this area. Non-Indigenous participants suggested that the psychology curriculum should incorporate increased exposure to lived experiences (28%), Indigenous-specific information (e.g. the impact of intergenerational trauma; 24%), more practical exercises (20%), and guidelines for adapting existing clinical interventions (28%). The study also revealed indicators of racially motivated biases in some participants’ responses.

**Conclusion:**

All Aboriginal and the majority of non-Indigenous participants reported that HE psychology training did not adequately prepare them to work with Aboriginal and Torres Strait Islander clients.

## Key points

**What is already known about this topic**

1. Psychology as a discipline has perpetuated harm towards Aboriginal and Torres Strait Islander peoples due to its colonial heritage.
2. Psychology higher education courses have typically taught a Westernised curriculum, often excluding Aboriginal and Torres Strait Islander content, however this is changing.
3. The current psychology workforce experiences a lack of preparedness to meet the needs of Aboriginal or Torres Strait Islander peoples.

**What this topic adds:**

1. All Aboriginal participants and the majority of non-Indigenous psychologists reported their higher education courses were inadequate in preparing them to work culturally responsively with Aboriginal and Torres Strait Islander clients.
2. Most non-Indigenous psychologists reported apprehensions about working with Aboriginal and Torres Strait Islander clients for the first time.
3. There is a need to build educators’ capacity to Indigenise the psychology higher education curriculum to better prepare the psychology workforce to work with Aboriginal and Torres Strait Islander clients.

## Introduction

Aboriginal and Torres Strait Islander peoples in Australia continue to resist the ongoing impacts of colonisation to ensure the continuation of culture. However, the ongoing impacts of policies and practices related to colonisation, including racism, mean Aboriginal and Torres Strait Islander peoples are more likely to experience poorer social and emotional wellbeing (SEWB) in comparison to their non-Indigenous counterparts (Dudgeon et al., 2020). Connection to culture is a vital ingredient of SEWB, and studies have found that being engaged and connected with culture has a beneficial impact on SEWB (Paradies, 2016; Waterworth et al., 2015). Despite this knowledge, healthcare services continue to apply a Western lens to supporting Aboriginal and Torres Strait Islander people, and this lack of cultural safety and responsiveness within Australian “mainstream” systems has resulted in further perpetuation of harm through racisms, misdiagnosis, continued mistrust, and further oppression (Clark & Hirvonen, 2022) (Durey & Thompson, 2012).

The Westernised discipline of psychology has contributed to the perpetuation of harm towards Aboriginal and Torres Strait Islander peoples. This harm has occurred through exploiting our oppression through pathologising the Indigenous experience and endorsing non-Indigenous, culturally biased psychological approaches, as well as failing to recognise and incorporate Indigenous knowledges and perspectives (Clark & Hirvonen, 2022). The Australian Psychological Society (APS) acknowledged the discipline’s contribution to the perpetuation of harm and the benefit that non-Indigenous psychologists gained from this:

“*Conducting research that has benefitted the careers of researchers rather than improved the lives of the Aboriginal and Torres Strait Islander participants*” (Carey et al., 2017). As such, the APS committed to reconsidering the approach in an apology issued in 2016 (Carey et al., 2017).

We, as psychologists, have not always listened carefully enough to Aboriginal and Torres Strait Islander people. We have not always respected their skills, expertise, world views, and unique wisdom developed over thousands of years. Building on a concept initiated by Professor Alan Rosen, we sincerely and formally apologise to Aboriginal and Torres Strait Islander Australians. (Carey et al., 2017)

The APS apology in 2016 helped to spearhead the call to include Aboriginal and Torres Strait Islander cultural responsiveness into the field of psychology (Dudgeon et al., 2020). However, without meaningful engagement and intent from the broader psychology discipline, the words in the apology may be considered just that; only words, and the symbol of another unmet promise to the Aboriginal and Torres Strait Islander peoples.

Only a year earlier, O’Connor et al. (2015) interviewed eight Australian non-Indigenous psychologists to identify the necessary professional and personal skills that enable effective psychological practice when working with Aboriginal and Torres Strait Islander clients. Having credibility, centring relationships, understanding contexts, taking a holistic approach and being flexible were reported as the keys to good practice (O’Connor et al., 2015). However, O’Connor et al. (2015) asserted that these factors were not at the time incorporated into the standard psychology curriculum in HE institutions, with the authors advocating for ongoing adjustments to the university curricula in order to graduate culturally responsive psychologists.

Historically, the legacy of Aboriginal and Torres Strait Islander peoples has been either missing or misrepresented within primary, secondary and HE in Australia (Bond et al., 2020; Brown, 2019; Gillian et al., 2017. University curricula has reproduced colonial epistemologies as the only necessary worldview, ignoring Aboriginal and Torres Strait Islander knowledges, perspectives, learnings, theories and methodologies (Bond et al., 2020; Gillian et al., 2017; Smith, 2023). This in turn has contributed to the continuation of the colonial project, entrenching education about Indigenous people through a deficit discourse (Vass, 2012).

In this context, a discourse is suggested to encompass thoughts that are communicated through physical writing and practices, or by verbal means which contribute to shaping ideas, concepts or materials that reinforce power inequalities (Fogarty et al., 2018). A deficit discourse is a discourse that positions individuals or groups in the frame of deficiency (Fogarty et al., 2018). In psychology HE courses, all too often we have heard how sick, disadvantaged, or in need Aboriginal and Torres Strait Islander peoples and communities can be, often disregarding the strength, resistance, healing, belonging and pride that can come from being part of the oldest living culture in the world. Including different strength-based cultural perspectives into the curriculum involves stepping outside of the monocultural view of psychology and psychological wellbeing and recognises that holding on to such views and practices continues to privilege the dominant social group at the expense of the inclusion of psychologically effective and informative knowledges and perspectives of marginalised groups (Moncrieffe et al., 2020).

Without the inclusion of critical Indigenous knowledges and perspectives in psychology training, it is likely that many non-Indigenous psychologists may struggle to understand the challenges and protective factors of Aboriginal and Torres Strait Islander clients, reducing their capacity to provide effective and culturally responsive support. This disconnection can lead psychologists to perceive their “monocultural” training as “the norm”, - the idea of “mainstream” psychological care – in turn, further perpetuating psychologists’ limited competencies to work with, and unhelpful attitudes towards Aboriginal and Torres Strait Islander clients (Wright et al., 2021). However, HE can be an effective tool to challenge and dismantle students’ biases prior to graduation and professional practice with clients.

Fortunately, although in its infancy (first iteration began in 2013), a national movement to address the whitewashed psychology curricula is occurring across Australian HE institutions, with projects like the Australian Indigenous Psychology Education Project (AIPEP) headed by Professor Pat Dudgeon, a Bardi woman from the Kimberley region in Western Australia. AIPEP, now in its second iteration, works towards increasing cultural responsiveness in psychology HE and the mental health workforce, as well as increasing the number of Aboriginal and Torres Strait Islander psychology students who graduate (Ohan et al., 2023).

In 2016, AIPEP responded to the recognition within HE that Aboriginal and Torres Strait Islander content had been conceptualised as an “add-on”, to the existing curricula (Dudgeon et al., 2016). AIPEP created three frameworks. One to guide educators to integrate Indigenous knowledges and perspectives into the psychology curriculum with the aim to enhance the cultural responsiveness of psychology graduates; the second to guide stakeholders towards retaining and supporting Aboriginal and Torres Strait Islander psychology students; and the third to build the capacity of the current psychology workforce to practice safely and effectively with Indigenous clients (Dudgeon et al., 2016).

Similar work in the cultural responsiveness space is being conducted by Indigenous-led organisations like Indigenous Allied Health Australia (IAHA). IAHA is a national, member-based organisation that, amongst other things, supports Aboriginal and Torres Strait Islander allied health students and graduates, and contributes to building a culturally safe and responsive allied health workforce (Cranney, 2015). Due to these Indigenous-led initiatives and the relentless disruption from Aboriginal and Torres Strait Islander peoples and our allies in the HE institutions, we are seeing change. Most notable for psychology, is the work of AIPEP.

Smith et al. (2024) have recently developed and validated a Cultural Responsiveness Assessment Measure (CRAM), which they describe as a self-reflection tool for mental health practitioners and students to evaluate their culturally responsive practices when working with Aboriginal and Torres Strait Islander peoples. A model of cultural responsiveness for mental health professionals underpins the CRAM, which centres reflexivity and incorporates nine surrounding domains: awareness, knowledge, inclusion, relationships, cultural respect, cultural safety, social justice/human rights, self-reflection, cultural humility, and cultural competencies (Smith et al., 2024). The CRAM has demonstrated good face and construct validity, strong overall internal validity and test–retest reliability (Smith et al., 2024). This tool can serve as a reliable instrument to introduce into the psychology HE curricula so that students can become familiar with reflective practice and the elements of cultural responsibility. Further, the nine underlying domains can serve as a starting point for educators to explore and learn ways to incorporate them throughout the psychology subjects.

### New accreditation standards to assess student competencies in cultural responsiveness

There has been a call for more culturally responsive psychologists, leading to a review and update of the accreditation standards for HE psychology courses. The Australian Psychology Accreditation Council (APAC) responded to the good work that has been done to raise awareness of the gaps in HE psychology training. APAC, in collaboration with AIPEP, recognised:

Non-Indigenous students of psychology need to understand the historical context of colonization and the ongoing legacy of that history in a contemporary space. Potentially more important is understanding the role played by the discipline and profession of psychology in contributing to solutions to address the inequities faced by Aboriginal and Torres Strait Islander peoples. This requires reflexive analysis of self as well as of the knowledge base and practices of the discipline. (APAC, 2023, p. 4)

Therefore, in 2019, APAC introduced Criterion 3.8 in their psychology course accreditation standards which requires psychology HE providers to demonstrate how they assess student competencies related to cultural responsiveness (APAC, 2023). In 2021, APAC acknowledged that HE providers were finding it challenging to incorporate cultural responsiveness into their curricula as they did not understand the definition and purpose of Criterion 3.8. Further, they found that HE educators also did not know how to assess student competencies related to Criterion 3.8 and found many educators to not be culturally responsive themselves due to this Criterion being so newly mandated (APAC, 2023). This led to the formation of a Working Party, consisting of senior Indigenous and non-Indigenous psychology academics, who were tasked with exploring how best to provide guidance and support to educators in demonstrating alliance with Criterion 3.8, resulting in the creation of the “Annexure to the APAC evidence guide: Standard 3 Program of study criterion 3.8” (Bucks et al., 2023). This illustrated a genuine effort on behalf of APAC to support educators through this transition.

Another example of a national shift in this space is the Australian Universities Accord Final Report (Department of Education, 2024). The report created by the Commonwealth Department of Education presented 47 recommendations for HE reform, one being that Australia needs to establish a high-quality, equitable HE system (Department of Education, 2024), with five priority actions to operationalise this target. Two of the priority actions explicitly recommend ways to decrease inequity experienced by Aboriginal and Torres Strait Islander peoples within the HE system: “*First Nations people to be at the heart of Australia’s higher education system*” (Department of Education., 2024, p. 237).

Australian Health Practitioner Regulation Agency (AHPRA, 2024) regulates the psychology profession in Australia and is following suit. AHPRA recently updated the competencies needed for provisional psychologists to become registered as generalist psychologists and placed: “*a greater emphasis on professional reflection and reflexivity, purposeful and deliberate practice and self-care … to practise professionally and safely in a sustainable way*”. More specifically, competencies 7 and 8 have been “*enhanced to better address the diversity in the Australian community*” (AHPRA, 2024, Competency 7) and “*to emphasise culturally safe care with Aboriginal and Torres Strait Islander Peoples, families and communities*” (AHPRA, 2024, Competency 8).

I (the first author) am an Aboriginal woman coming to the closing of my 10-year university career as a psychology student. This paper was created in partial fulfilment to meet the requirements of the Doctor of Philosophy (Clinical Psychology). It was born due to my experiences across my several years of studying psychology degrees at several universities situated in Gold Coast, Sydney, and Melbourne. I found it remarkable the way in which Aboriginal and Torres Strait Islander peoples are taught about and talked about in the classroom. Like we were the problem and needed to be saved. Unfortunately, I have witnessed little improvement in the curriculum across the last decade. I often found myself taking on the colonial burden of educators within the classroom to educate my peers and teachers, challenging the harming narrative and discourses being taught, and advocating for change within the system. It is not by coincidence that my doctoral thesis focuses on incorporating Aboriginal and Torres Strait Islander culturally responsive practices in both psychological practice and HE. From my personal experiences and yarning with other mob studying psychology at HE institutions, I can confidently say that we need to adequately train future psychologists to be culturally responsive to the needs of Aboriginal and Torres Strait Islander clients if we are to work towards closing the mental health gap. Understanding the barriers faced by non-Indigenous psychologists in working with Aboriginal and Torres Strait Islander clients may be particularly important to address their general lack of preparedness.

Therefore, this study explores psychologists’ perspectives on the degree to which they feel their HE prepared them to work with Aboriginal and Torres Strait Islander clients. Furthermore, this study aims to contribute to the solution by gathering participants’ suggestions about how psychology HE training could be improved to better develop students’ cultural responsiveness when working with Aboriginal and Torres Strait Islander clients.

## Method

### Research design

This study employed an electronic mixed-method survey to explore psychologists’ perspectives about how well their HE courses prepared them to work with Aboriginal and Torres Strait Islander clients. Further, participants provided suggestions for improvements to the psychology HE curriculum. This study was part of a more extensive study exploring cultural responsiveness in psychology practice and HE. More details can be found in Darnett et al. (2024).

### Participants recruitment

The participant recruitment methods have been reported in great depth in the associated study (Darnett et al., 2024). However, briefly, participants were recruited using targeted social media and online platforms, as well as university affiliations through email invitations and poster advertisements. Prospective participants were informed that the research explored how psychologists supported Aboriginal and Torres Strait Islander client’s wellbeing. Participants followed the Qualtrics link in the recruitment materials.

Group allocation occurred to respect and privilege Indigenous voices and perspectives. More details about the group allocation method are available in supplementary file 1.1. This article mainly reports on the findings from Group 3 (non-Indigenous psychologists that have not worked with Indigenous clients). Due to Group 3’s lack of experience working with Aboriginal and Torres Strait Islander clients they were directed the questions exploring their HE experiences.

#### Mixed-method survey

This is the second paper published based on the data collected using this research methodology. Therefore, the questionnaire development, overall sample demographics and between-group differences have been previously reported (Darnett et al., 2024). Furthermore, Darnett et al. (2024) also explored the comparison between the study’s overall participant sample and the reported psychological workforce demographics in the Australian Health Practitioner Regulation Agencies (AHPRA, 2024) annual report 2022/2023. Outside of the demographic questions, additional questions posed to participants can be found in supplementary file 1.2.

### Data collection

The data collection process has been described elsewhere (Darnett et al., 2024). In brief, an anonymous Qualtrics electronic survey with both open and closed questions was distributed to collect qualitative and quantitative data from participants. Additional information can be found in supplementary file 1.3.

### Data analysis

The complete data analysis process has been documented elsewhere and for a complete exploration of the participants’ demographics and analysis, see Darnett et al. (2024). An inductive approach to conventional content analysis was employed on the open-ended qualitative responses provided by participants (Hsieh & Shannon, 2005; Vears & Gillam, 2022).

### Ethics

Ethics approval was granted by Swinburne University’s Human Research Ethics Committee (ref: 20235840–14219). Further information pertaining to the ethics can be found in supplementary file 1.4.

## Findings

### Participant demographics

One hundred and eight people comprised the final sample: 89 females, 18 males, and 1 non-stated gender. Of the 108 participants, 15 identified as Aboriginal, 93 reported to be non-Indigenous, and the ages ranged from 22 to 83 years (*M = 40.7, SD = 12.84*). See supplementary file 2.1 for the demographics of each group post allocation. The participants’ demographics were originally published in the linked study (Darnett et al., 2024). With permission from the journal, the demographics table has been replicated and can be viewed in supplementary file 2.2. Most notably, there was a large difference identified between groups in the registration-type category. Groups 1 and 3 comprised a greater percentage of provisional psychologists than Group 2.

### Participant preparedness

**Figure 1.**
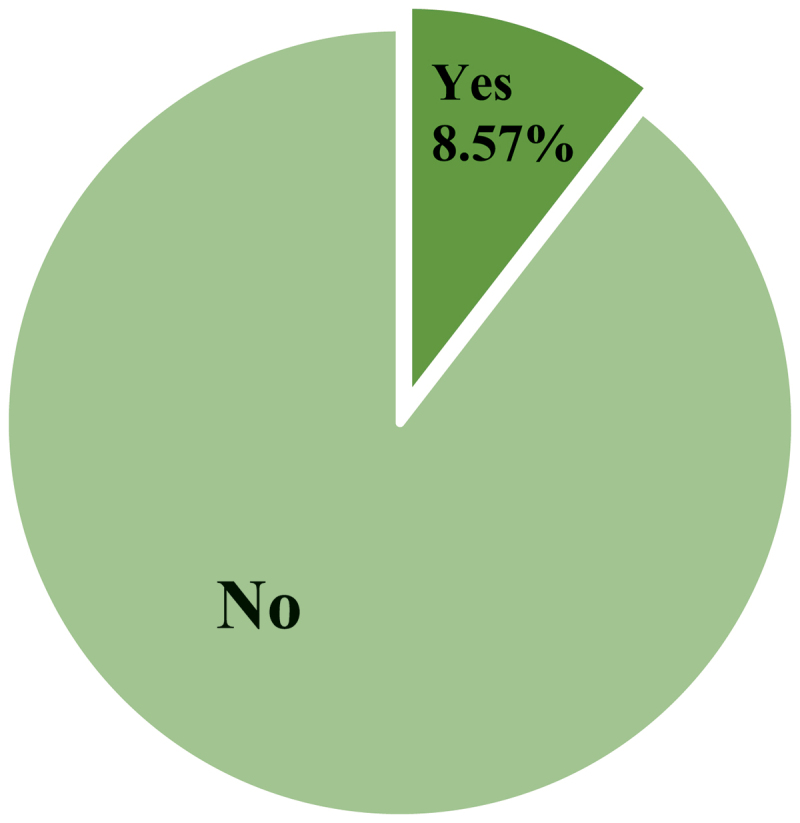
Participants’ perspectives if their psychology higher education training adequately prepared them to work with Aboriginal and Torres Strait Islander Clients.

All participants (*N* = 108) were asked if, from their perspective during their tertiary education, they and their peers were provided with enough education to adequately adjust their psychological practice to work with Aboriginal and/or Torres Strait Islander clients. Overall, 91.43% (*n* = 105, including all Aboriginal participants) reported feeling that their HE training inadequately prepared them to work with Aboriginal and Torres Strait Islander clients. Figure 1 depicts the results.

(*n* = 105, missing = 3)

All 9 participants who indicated they had been provided enough education were non-Indigenous (Group 2 *n* = 7, 4 females, 3 males; Group 3 *n* = 2, 1 female, 1 male). Interestingly, male psychologists comprised 16.8% of the total participant sample and yet accounted for approximately 44.5% of participants who indicated that their HE in psychology adequately prepared them to work with Aboriginal and Torres Strait Islander peoples.

Overall, the majority of participants agreed that there was minimal training provided by their HE institutions. All participants were provided the opportunity to explain their responses. Participants from all groups commonly suggested that some universities’ attempts to educate students about culture have been unsuccessful and, at times, offensive.

These sentiments are evidenced by Participants 8 and 54 in Group 3 who wrote “*sometimes seemed like an ‘add on’ without the chance to delve deeply*” and “*I don’t feel the training was sufficient and I still don’t feel confident to work with this demographic*”, respectively. Participants in Group 2 had similar comments reporting “*I can’t recall a single lecture let alone a course or placement dedicated to this. Profoundly inadequate*” (Participant 74). As well as “*we had a two-day workshop that was offensive and highly inadequate. If I took at face value what I felt they were pushing, I would have believed I should never try to support Indigenous clients because we could never comprehend them sufficiently to have even the most fundamental understanding*” (Participant 65).

Finally, and possibly most concerningly, Participant 29 who is an Aboriginal psychologist from Group 1 reported:

The so-called “Cultural Awareness” 1-day workshop was traumatising as an Aboriginal person. I had my identity invalidated by 12 of the 13 other students who attended at the start of the course and the facilitators were not even Psychologists or knowledgeable about culturally competent practices within the clinical/therapeutic setting - so much that I complained to have the workshop ceased. The School of Psychological Science then liaised with the School of Indigenous Studies to engage an Indigenous Clinical Psychologist with appropriate knowledge. I was my uni’s first Indigenous Postgraduate student - the cohort in year before had complained about the workshop and one facilitator was removed. One of the 2 facilitators in my workshop said “Suicide is just a call for help”…… I received a number of micro/macro aggressions from my student peers - so 100% no they are not equipped. For example, on my first day in postgrad one insisted it was ok for her to have female participants learn the didgeridoo and told me I was wrong when I advised her it was culturally inappropriate.

More qualitative responses can be found in supplementary file 2.3

### Participants’ apprehensions

Participants in Group 3 (*n* = 20) were asked if they held any apprehension about working with Aboriginal and Torres Strait Islander clients for the first time. Out of the total 16 responses, two people (12.5%) indicated they did not hold apprehensions, reporting “*they are individuals, like all other clients*” (Participant 33, Group 3), and “*I’d like to think my approach to working with clients is accommodating to all and can be (and is) adjusted to work best for my individual clients*.” (Participant 14, Group 3).

Of the 14 participants in Group 3 (87.5%) who reported holding apprehensions about working with Aboriginal and Torres Strait Islander clients for the first time, many provided qualitative responses that suggested the apprehension was due to their limited understanding of cultural differences *“having a limited understanding of different cultures and practices. Limited knowledge of additional requirements such as retention of records”*. (Participant 8, Group 3) and *“my understanding is that First Nations people have a somewhat different belief system regarding mental ill-health and I’m not confident our western tools for psychological treatment are appropriate”* (Participant 9, Group 3).

While other participants questioned their capabilities suggesting they had a “*lack of experience and training*” (Participant 44, Group 3), and “*I have heard it can be difficult to be accepted as a practitioner, and this role requires a lot more rapport building*” (Participant 63, Group 3), and “*I have done some training and quite a bit of reading but worry that I would not be competent*” (Participant 70, Group 3). It was found that some participants were worried about perpetuating harm, “*I am apprehensive to work with any client for the first time, especially ones that are from diverse backgrounds as I am worried, I may say something inadvertently (through my own ignorance) that may be taken to be offensive*” (Participant 12, Group 3), and “*mainly, I would be afraid that I might inadvertently perpetuate historical wrongdoings. I want to create safe spaces and my apprehension would be driven from worry that if I was not successful that I might perpetuate harms*” (Participant 28, Group 3). These findings are important as they signify a recognition of the power a psychologist holds, as well as the harm that can be done when working clinically with clients.

### Participants’ suggestions toward improving higher education psychology courses

Group 3 (Non-Indigenous Psychologists with no experience with Indigenous clients) were asked a follow-up question to contribute their perspectives regarding information they would find helpful to guide their psychological practice with Aboriginal and/or Torres Strait Islander clients. From the content analysis, participants responses could be categorised under four headings (*Lived Experience, Education, Practice Exercises, Intervention Guidelines*). Participants reported they wanted access to more Indigenous peoples with *Lived Experience;* a deeper *Education* of Indigenous history and the implications this has for psychology; more *Practice Exercises* to apply their understanding; and specific *Intervention Guidelines* informing the adaption of therapies. Themes and example quotes are available in supplementary file 2.4.

### Participants’ plans to increase cultural knowledge

**Figure 2.**
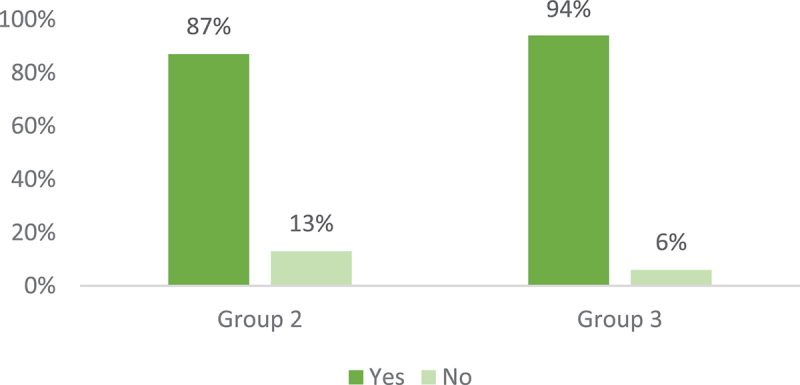
Percentage of non-indigenous participants planning to increase their knowledges and understanding of Aboriginal and Torres Strait Islander culture by group.

Participants from Groups 2 and 3 were asked if they planned to increase their knowledge and understanding of Aboriginal and Torres Strait Islander culture. The majority of participants (90.5%) indicated they planned to undertake further professional development in this area (see Figure 2). This illustrates psychologists’ desire for culturally responsive education to allow them to practice more effectively with Aboriginal and Torres Strait Islander clients.

(Group 2 non-Indigenous psychologists with experience working with Aboriginal and Torres Strait Islander clients *n* = 69, Group 3 non-Indigenous psychologists with no experience working with Aboriginal and Torres Strait Islander clients *n* = 16)

Both Groups 2 and 3 were asked to provide examples of how they plan to increase their cultural knowledge and understanding. There were five themes that emerged from the content analysis, which were, *Supervision, Workshops or conferences, Participate in Indigenous focused activities, Learning from Indigenous peers or clients, and Exploring academic research and grey literature*. Supplementary file 2.5 contains some examples of the participants’ responses.

### Participants’ cultural questions

Participants from both Groups 2 and 3 were also asked if they had any specific cultural questions that they would like answered regarding psychological practice with Aboriginal and/or Torres Strait Islander client/s. Some of the responses would be easily addressed through an internet search, for example, “*what language/terminology is preferred, and are there aspects of standard psychological practices that might be ill-fitting or mismatched to culture?*” (Participant 8, Group 3), “*how do these preferences differ between First Nations populations, noting that, for example, the Wurundjeri people are different from the Ngunnawal people*.” (Participant 9, Group 3). Lastly, “*how do I treat an ATSI client differently from all clients? I am unsure if I need to do different things to be culturally aware when I feel I already do so for every client and don’t discriminate positively or negatively based on culture*” (Participant 48, Group 2).

Some participants had specific questions about the practical aspects of psychology, like how to adapt interventions and culturally appropriate psychometrics. For example, Participant 23 in Group 2 said: “*I think I have good training and understanding in history, experiences of Aboriginal and/or Torres Strait Islander clients, etc etc but very little training in practically, how to adapt practice to be more culturally safe (e.g., what do I actually do in the session)*”, Participant 60 in Group 2 asked “*how mainstream psychotherapeutic practices are best adapted to assist Aboriginal clients. How this can be taught. How such resources can be built*”, while Participant 37 in Group 3 echoes these sentiments and said, “*how best to use/adapt psychological knowledge to best support them*”. Participants 74 and 81 in Group 2 were focused on neuropsychology assessment tools, “*I would very much like a framework for neuropsychology to work with Aboriginal and Torres Strait Islander peoples*”. and “*culturally appropriate neuropsychology assessment”* respectively. Participant 108 in Group 2 also agreed that *“more information on the validity and utility of neuropsychology tests in this population would be helpful*.”

Other participants asked more culturally specific questions; for example, Participant 10 in Group 2 said, “*I would like to know more about cultural practices like men’s business and women’s business*, *etc. It’s like the practices are named but not explained*”. Participant 25 in Group 2 added, “*more about things that actually show up in the room, like family structures and gender roles and ways to build or explore identity as an Aboriginal or Torres Strait Islander person. Ways of healing, ways to approach elders, culturally normative experiences that could be mistaken for mental ill-health*”. Participant 45 in Group 2 added, “*I think I would like to be told clearly “this is how you ask someone about their dreaming/totem, cultural healing practices, etc*”.

Group 2 were curious about more existential questions, with Participant 11 asking,

how can a ‘white person’ ever hope to be able to provide meaningful therapy when there is such a divide. It feels like appropriation to use words like ‘mob’ or try ‘yarn’. We are taught these words and even suggested that we try yarning, but how do we do that in a way that is respectful rather than inappropriate?

*Also, participant 56 questioned, “*when will universities stop pretending that Western psychology, and particularly psychiatry is, without significant change, appropriate for Aboriginal people?”

Lastly, some participants in Group 2 asked questions that were specific. For example, Participant 62 asked, “*how to combat racism from other clients?*”. While, Participant 73 requested information about,

how to show I’m different? I’d like to be able to make it clear that I’m a supporter and an ally, but I worry about touting myself because I know so many people say one thing but do another, and so many of my clients experience that before they get to me.

Participant 59 added, “*why perpetrators of (Domestic and Family Violence) DFV are often protected by the community and the survivor is often further abused for trying to leave*.” Participant 84 was curious, “*how to deal with the normalisation of violence, abuse in communities*”. Supplementary file 2.6 has a more extensive list of questions qualitative responses.

### Participant biases

The implicit and explicit bias apparent in the qualitative responses indicates that some psychologists may not be adequately trained to work with Aboriginal and Torres Strait Islander clients. On the flipside, several participants displayed an understanding of the need to be conscious of their biases. For example, in response to being asked to explain why they think psychologists need to adjust their practice when working with Aboriginal and Torres Strait Islander clients Participant 13 stated:

yes, we need to be aware of what is culturally appropriate as we would with any client group. However, for Aboriginal and Torres Strait Islander clients I think it is particularly important to be aware of the ways in which health services and particularly psychology may have been used to further disadvantage them, and the complex interactions that we as clinicians may be part of when supporting someone who is Aboriginal and Torres Strait Islander in health services. We also need to be aware of our own implicit biases and aware that much of what we have been taught does not necessarily hold true for Aboriginal and Torres Strait Islander people, as many of our historical research has been racially biased or has excluded other perspectives.

(Group 2, Participant 13)

Participant 85 in Group 2 stated, “*potentially; like all clients there is no one size fits all – collectivist culture, respect for elders, need to be present for significant events, past lost opportunities due to inequities and biases*”. Further, Participant 87 in Group 3 wrote, “*we need to check our own biases and assumptions. We also need to understand that our training was delivered through a white/western lens and cannot assume that this is therefore appropriate for all backgrounds*”.

Other participants displayed their consciousness of biases when asked to explain any challenges they have experienced when Aboriginal and Torres Strait Islander clients by statin*g, “being more conscious or my own bias has been challenging and continues to be. Meaning I have to make less assumptions and the client is in much more of the driver’s seat…*” (Participant 106, Group 2). While Participants 70 in Group 3 suggested “*decolonisation training. Building awareness of implicit bias. Training in appropriate communication, measures & therapeutic approaches*” when responding to the question that asked what information they would find useful to guide their psychological practice with Aboriginal and Torres Strait Islander clients. However, without explicitly asking about participants’ understanding of bias, we cannot report on the participants understanding of their biases.

All participants responses, besides one, were interpreted by the research team to contain little to no intention to overtly display their biases and so some responses were considered to contain implicit bias. However, the following examples of bias was perpetuated by the same member of Group 2 who stated, “*I treat all people like ‘people’. Aboriginal identity is no more unique than any other cultural subset*”; and “*honest truth – most people will never actually need to know this because Aboriginal people make up 3% of Australia’s population – i.e., they are not a majority of the workload*”; and “*I prefer to use my professional development time upskilling in things that can help ALL of my patients – not focusing on thangs that make a small difference to a very small minority*”; and ”*I had a family member who tried to demand information about someone that I was supporting. They said [very mistakenly] their cultural protocols could override my patient’s privacy protections. The family member was very aggressive. I was very close to calling the cops to have them escorted out*” (Participant 39, Group 2).

These responses from Participant 39 were considered to be explicit bias due to the conscious nature of the deficit perception of Aboriginal and Torres Strait Islander peoples and an attitude that minimised the need to upskill to provide a culturally safe experience for Aboriginal and Torres Strait Islander clients.

## Discussion

Overall, this study’s findings highlighted that psychology HE training did not adequately prepare participating psychologists to work with Aboriginal and Torres Strait Islander clients. This is evidenced by their self-reported apprehensions, as well as evidence of biases that were found in participant responses during analysis. These biases give way for more harm to be perpetuated on Aboriginal and Torres Strait Islander peoples, which in turn contributes to the increased mental health disparities that we experience. To address these biases, participants presented many ideas about improving the curricula to develop psychologists’ cultural responsivity when working with Aboriginal and Torres Strait Islander clients.

The majority of participants in Group 3 (non-Indigenous psychologists who had not worked with Aboriginal or Torres Strait Islanders previously) indicated that they held apprehensions about working with Aboriginal and Torres Strait Islander clients for the first time. Participants reported that they feel unequipped to work with Aboriginal and Torres Strait Islander peoples because of their lack of cultural knowledge. Participants were concerned that they might inadvertently perpetuate more harm, which is a legitimate concern, as a lack of cultural knowledge on behalf of the psychologist is a real risk for Aboriginal and Torres Strait Islander people’s mental health and wellbeing. Participants’ answers suggest that the respondents understand the power they hold as psychologists, and are willing to engage in critical self-reflection to identify personal gaps in their knowledge and abilities aligning with Smith et al., (2024) reported findings that emphasised the necessary capabilities of a culturally responsive mental health professional.

Biases were present in some participants’ responses. The presence of biases in the qualitative responses provides further evidence that current HE psychology training is insufficient at developing some student’s reflexivity and insight which can be harmful for Aboriginal and Torres Strait Islander clients. However, the majority of the participants showed motivation towards providing a culturally safe psychological service for Aboriginal and Torres Strait Islander clients. For example, 87.5% of participants reported holding apprehensions about working with Aboriginal and Torres Strait Islander clients for the first time, with one of the main reasons being a fear of perpetuating harm. This fear results from deficits in the psychology HE training, in that 91.43% of the participants reported that their HE training in psychology did not adequately prepare them to work with Aboriginal and Torres Strait Islander clients.

As custodians of the world’s oldest living culture, Aboriginal and Torres Strait Islander peoples possess a wealth of wisdom and knowledge that can enrich the discipline of psychology. These findings reveal that Indigenous knowledges, perspectives and SEWB resources are missing from psychology HE training that may be useful to enhance all people’s mental health and wellbeing. The rationale for HE psychology courses to make space for Indigenous psychology perspectives is strengthened as more empirical evidence emerges from outside of the psychology discipline, supporting the applicability of Indigenous models for non-Indigenous populations having positive outcomes (see, Jiao et al., 2009; Throsby & Petetskaya, 2016; Wilson, 2009).

To work towards improving the current psychology HE curricula, participants suggested they would like more exposure to Aboriginal and Torres Strait Islander people and to hear from lived experiences. They also suggested that they would like to learn more about intergenerational trauma and how it continues to impact Aboriginal and Torres Strait Islander clients. Participants also acknowledged they would like to engage in practical exercises that include critical self-reflection to explore biases, appropriate assessment tools and psychometrics for Indigenous clients, and communication techniques. Finally, participants suggested that manualised guidelines of how to adapt interventions to be more culturally responsive when used with Aboriginal and Torres Strait Islander clients would be useful.

The elements highlighted by participants as to what they thought might enhance their cultural responsiveness (e.g., understanding historical impacts and awareness of bias) overlapped with suggestions in the AIPEP curriculum framework (Dudgeon et al., 2016) as well as the CRAM, and domains of cultural responsiveness (P. Smith et al., 2024). Moreover, none of the participants’ responses aligned with O’Connor et al’s (2015) findings that credibility, centring relationships, understanding contexts, taking a holistic approach and being flexible are important when practicing with Aboriginal and Torres Strait Islander clients. Identifying these additional gaps further supports to the main findings of this study, being that HE is not adequately training future psychologists to work with Aboriginal and Torres Strait Islander clients.

Most participants (90.5%) indicated that they plan to engage in additional training to increase their knowledge and better understand Aboriginal and Torres Strait Islander culture, demonstrating a great desire from psychologists to learn. Participants’ growing willingness to learn should be celebrated and reinforced by including Indigenous knowledges and perspectives into psychology HE. Furthermore, integrating Aboriginal and Torres Strait Islander knowledges and perspectives into psychology higher education curricula may foster greater feelings of respect and belonging for Aboriginal and Torres Strait Islander students. This, in turn, may positively contribute to increasing the numbers of Aboriginal and Torres Strait Islander psychologists and psychology academics who are in high need (Dudgeon et al., 2016; Ohan et al., 2023). Collectively, this creates an even stronger rationale for educators to include Aboriginal and Torres Strait Islander knowledges and perspectives into the psychology curriculum.

Participants indicated a number of ways to increase their knowledge, including through supervision, workshops or conferences, participation in Indigenous activities, academic and grey literature, and finally learning from their Indigenous peers or clients. Two concerns are highlighted by participant responses. Consulting academic literature was the least mentioned strategy by participants for improving their understanding of culturally responsive practices. This could be interpreted as a gap in the type of information seeking that one would expect of a university graduate, especially a psychologist whose competence to practice should be evidence-based and informed by the scientist-practitioner model (Jones & Mehr, 2007) that expects the profession to consult high-quality academic literature. Further limitations of the scientist-practitioner model include a bias to centre whiteness and subjectiveness to publication bias (Dudgeon et al., 2016; Luke et al., 2022; Parter et al., 2021). HE institutes and psychology educators may benefit from expanding beyond scientific databases for educational resources. Adding more culturally grounded and responsive resources into psychology subjects’ reading lists could start to bridge a gap, as would evidence-based professional development for psychologists (Hutchings et al., 2019).

Forty-eight percent of respondents suggested that they would learn from their Indigenous clients or peers. Non-Indigenous psychologists need to ensure that they are not just shifting the colonial burden onto their Indigenous peers to educate them. It is important to think critically about the knowledge and insight being requested from Indigenous peers, as it is not our job to educate our colleagues. A similar sentiment can be applied to Indigenous clients, as we are not there to educate non-Indigenous psychologists about our culture and how it impacts worldviews; this is the responsibility of the HE institutions and the individual practitioners. It is important for non-Indigenous psychologists and educators to revisit the commitments made in the APS (2007) apology, and action them as meaningfully and regularly as possible.

The cultural questions that participants asked were diverse, reflecting the varied levels of cultural responsiveness and knowledges held by psychologists. Some straightforward questions about terminology were posed suggesting some psychologists may not have a basic understanding of Aboriginal and Torres Strait Islander cultures which is of concern, and at the same time reinforces the rationale for this study. The more fundamental questions reported by participants (e.g., correct terminology) could be a starting point for first-year psychology educators to introduce an appropriate terminology guide. Moreover, it could be a consideration for tertiary and primary school educators to consider developing students’ understanding of our culture earlier. There were more practical topics raised in the questions that educators could incorporate across the psychology subjects and degrees, increasing in complexities as the students’ experiences increase. Finally, not all questions posed by participants had easy straightforward answers, and responses may differ based on community place. Therefore, psychologists and psychology educators need to be equipped with the necessary skills to independently find the answers to questions about culturally responsive practice.

Reflecting on power dynamics as an element of cultural responsiveness for mental health professionals (P. Smith et al., 2024). The findings revealed males were more likely to endorse a statement of competence to work with Aboriginal and Torres Strait Islander clients than females. Male psychologists comprised 16.8% of the total participant sample and yet accounted for approximately 44.5% of participants who indicated that their HE in psychology adequately prepared them to work with Aboriginal and Torres Strait Islander clients. These findings have partially been supported in a systematic review undertaken by Vajapey et al., (2020) who found male medical trainees and health professionals self-reported higher levels of clinical knowledge, skills, procedural confidence, operative experience, and other competencies = in comparison to their female counterparts. This is despite no evidence to support differences in actual performance or skills between men and women at any level of training in medicine. This finding and the potential implications it has both broadly and within an Aboriginal and Torres Strait Islander context warrants further research.

Within the context of Australian psychology HE, more needs to be done to address the systemic racism that is highly concentrated at the intersection of the discipline of psychology and HE institutions to develop future psychologists’ general awareness of the Indigenous experience. The Carey et al. (2017) made their apology to Aboriginal and Torres Strait Islander peoples over 8 years ago. This highlights a disappointing truth; as a discipline, psychology appears to be committed to creating change, however in practice this commitment is actioned by the few, leaving Indigenous communities once again bearing the burden of empty promises. Since the APAC (2023) accreditation standard change came into effect in 2019, the small amount of action is particularly clear as many participants in Group 3 were provisional psychologists currently at different stages of their psychology HE training. In theory, after 5 years, the current students should be experiencing the benefits of innovation that currently exist. Therefore, it would be beneficial for future research to explore the differences in cultural responsiveness of psychology graduates based on the year of graduation.

To make meaningful changes, this needs to be approached from several directions. Aligning with the commitments suggested in the Universities Australia’s Indigenous Strategy 2022–2025, including developing Indigenous-specific SEWB-enhancing strategies to promote change, could also act as a useful tool to facilitate reform (Universities Australia, 2022). Further reforms are necessary to the discipline of psychology and the HE institutions to address the systemic and individual barriers perpetuated onto minority groups including Aboriginal and Torres Strait Islander clients. Therefore, the research team has constructed some recommendations based upon the outcomes of this paper to help facilitate change across the psychology discipline and HE institutions. Key recommendations that have come from this work are reported in supplementary file 3.

### Limitations

Some of the methodological limitations that have been previously identified (see Darnett et al., 2024) and are also relevant to this paper include no Torres Strait Islander representation, the parameters of group allocations needed further consideration, and the overrepresentation of provisional psychologists. The absence of Torres Strait Islander representation in this study means the application of these findings to this group should be carefully considered. Additionally, the parameters used for group allocation resulted in a lack of Indigenous voices and perspectives on the psychology HE curriculum. Furthermore, it resulted in a small sample size to draw upon. Undertaking this study again with only Aboriginal and Torres Strait Islander psychologists and psychology students could provide more nuanced insights and suggestions to improve the current HE curriculum.

This study’s large representation of provisional psychologists (50% of Group 3) should be noted. The study depicts psychologists who are apprehensive and uncertain of their abilities. This is often the case for most provisional psychologists as we continue through our HE degrees, gathering theoretical knowledge and starting to undertake placements in the workforce. However, this is likely an inaccurate indication of the psychology workforce as a whole. AHPRA requires psychologists to engage in continued professional development to maintain our registrations, and often, as our exposure to diverse clients and presentations increases, so do our abilities and confidence levels.

It was previously noted that the research team are limited by the methodological design in regard to examining participants’ responses for biases. The participants were not explicitly asked about their biases or their intent that contributed to these themes, and therefore this may not be an accurate reflection of the participants’ intent or levels of biases. Therefore, we acknowledge that this theme was only lightly engaged with from an Indigenous perspective and could only be suggested and not confirmed. Based on our interpretations in this article, consideration should be given to the potential for participant biases representing broader attitudes within the psychology profession.

### Future implications

A strong implication from this research is for universities and psychology educators to invest in incorporating Aboriginal and Torres Strait Islander knowledges and perspectives into the curriculum to enhance the cultural responsiveness capabilities of psychology graduates. To do so, universities need adequate resourcing for educators to engage in the learning journey and feel confident in their ability to teach the redeveloped curriculum. Community consultations and partnerships should be integrated into the psychology HE curriculum to expose students to diverse populations, worldviews, and opinions on wellbeing. This would facilitate sharing learnings from people with different lived experiences and becoming familiar with undertaking critical self-reflection and identifying biases. Lastly, a pathway for practising psychologists to gather cultural information to support their Aboriginal and Torres Strait Islander clients is needed to ensure clients receive a culturally safe and effective psychological service.

The potential presence of biases among the psychology workforce could have detrimental implications for Aboriginal and Torres Strait Islander clients. It was hoped, that by highlighting these biases, psychology educators might be compelled to address this through reforming the psychology curriculum. Due to the methodological limitations to explore bias present in this study, this should be explored in future research. However, the result of this study provides a snapshot of the current psychology workforce, that emphasises the historic and current gaps within psychology HE training that need to be addressed.

## Conclusion

Overall, participants, including all Aboriginal psychologists in the sample, reported that HE institutions are not adequately trained to work with Aboriginal and Torres Strait Islander clients. The findings indicate HE institutes and educators need to incorporate more culturally responsive content into the curriculum. Doing so is crucial in developing future psychology students’ abilities to work in a culturally responsive manner with Aboriginal and Torres Strait Islander clients. This in turn will likely contribute to improving Aboriginal and Torres Strait Islander SEWB.

## Supplementary Material

Supplemental Material

## Data Availability

The participants of this study did not give written consent for their data to be shared publicly, so due to the sensitive nature of the research, supporting data is not available.
