# Supplementary material for: Toward culturally responsive psychology higher education courses: psychologists’ perspectives on preparedness to work with Aboriginal and Torres Strait Islander clients
Source: Aust J Psychol. 2025 Mar 12;77(1):2474546. doi: 10.1080/00049530.2025.2474546 (PMC12218482; doi:10.1080/00049530.2025.2474546)
Supplement: Supplemental Material [file RAUP_A_2474546_SM0834.docx]

**Supplementary File**

**Table of Contents**

[1. Extension of Method Information 37](#_Toc537666686)

[1.1 Group Allocation Method 38](#_Toc138343450)

[1.2 Electronic Survey 38](#_Toc1753035484)

[1.3 Data Collection 38](#_Toc1350769672)

[1.4 Ethics 38](#_Toc2069243527)

[2. Extension of Findings Information 38](#_Toc1870538884)

[2.1 Participant Group Allocations 39](#_Toc1244560269)

[2.2 Participant Demographics 39](#_Toc374815397)

[2.3 Qualitative Responses Exploring Participant Perspectives Toward Higher Education Preparedness to Work with Aboriginal and Torres Strait Islander Clients. 40](#_Toc1951931412)

[2.4 Additional Qualitative Responses Exploring Participants Suggestions Toward Improving Psychology Curriculum 42](#_Toc102952478)

[2.5 Additional Qualitative Responses Exploring Non-Indigenous Participants Responses Toward Develop their Cultural Knowledge 43](#_Toc348402419)

[2.6 Additional Qualitative Responses Exploring Non-Indigenous Cultural Questions 44](#_Toc90901518)

[3. Key Recommendations 45](#_Toc271117733)

**1. Extension of Method Information**

**1.1 Group Allocation Method**

If the participants identified as Aboriginal, they were allocated to Group 1. If participants indicated they were non-Indigenous and currently or had previously worked in a psychological capacity with Aboriginal and Torres Strait Islander clients, they were allocated to Group 2.

Participants who identified as non-Indigenous and indicated they had not engaged in work as a psychologist with Aboriginal and Torres Strait Islander clients were allocated to Group 3. As Group 3 participants did not have experience working with Aboriginal and Torres Strait Islander clients and could not contribute meaningfully to providing their perspectives in psychological practice, the questions asked of this group were altered to focus on their psychology higher education experiences.

**1.2 Electronic Survey**

The questions posed to all participants included,

1. “During your tertiary education, do you feel you and your peers were provided with enough education to adequately adjust your psychological practice to work with Aboriginal and/or Torres Strait Islander clients? Please explain your answer.”

Questions posed to only Groups 2 and 3 were done so to respect the Aboriginal participants knowing, these included,

1. “Do you have any specific cultural questions you would like answered in your formal or informal professional development regarding psychological practice with Aboriginal and/or Torres Strait Islander client/s?”
2. “Do you plan to increase your knowledge and understanding of Aboriginal and/or Torres Strait Islander culture? Please explain your answer.”

The additional questions posed only to participants allocated to Group 3 included,

1. “Do you have any apprehension about working with Aboriginal and/or Torres Strait Islander clients for the first time? Please explain your answer”
2. “What information would you find useful to guide your psychological practice with Aboriginal and/or Torres Strait Islander clients?”

**1.3 Data Collection**

For a complete overview of the data collection process see Darnett and colleagues (2024). However, briefly, an anonymous Qualtrics electronic survey with both open and closed questions was distributed to collect qualitative and quantitative data from participants. The inclusion of open-ended responses allowed participants to elaborate on their numerical responses, and aligns with Indigenous research methods, and deepens the research teams understanding of participants’ perspectives, attitudes, and contexts. The quantitative results were exported to SPSS for analysis, while the open-ended responses were analysed using Microsoft word and excel programs.

**1.4 Ethics**

Ethics approval was granted by Swinburne University's Human Research Ethics Committee (ref: 20235840-14219). No Aboriginal ethics council was consulted during this process; however, the majority Aboriginal research team ensured careful oversight by drawing on their expertise. Further, the Swinburne ethics committee consulted Aboriginal and Torres Strait Islander experts when reviewing an ethics application aimed at conducting research within an Aboriginal and Torres Strait Islander population, which provided another layer of cultural guidance.

**2. Extension of Findings Information**

**2.1 Participant Group Allocations**

After group allocation took place, all 15 Aboriginal psychologists were placed in Group 1 (Aboriginal Psychologists), 73 participants were allocated to Group 2 (Non-Indigenous Psychologists with experience with Indigenous clients), and 20 participants were placed in Group 3 (Non-Indigenous Psychologists, with no experience with Indigenous clients).

**2.2 Participant Demographics**

|  | **Group 1 Aboriginal Psychologists**  **(n = 15)** | | **Group 2 Non-Indigenous Psychologists, with experience with Indigenous Clients**  **(n =73)** | | **Group 3 Non-Indigenous Psychologist, with no experience with Indigenous clients**  **(n = 20)** | | **Total Sample**  **(n=108)** | |  |
| --- | --- | --- | --- | --- | --- | --- | --- | --- | --- |
|  | *n*          % | | *n*% | | *n               %* | | *n* | *%* |  |
| **Gender**  Female  Male  *Missing* | 13  2  0 | 86.7  13.3 | 59  13  1 | 81.9  18.1 | 17  3  0 | 85  15 | 89  18  1 | 83.2  16.8 |  |
| **Country of birth**  Australia  Overseas  *Missing* | 15  0  0 | 100  0 | 55  17  1 | 76.4  23.6 | 14  6  0 | 70  30 | 84  23  1 | 78.5  21.5 |  |
| **Main Language**  English  Other | 15  0 | 100  0 | 71  2 | 97.3  2.7 | 20  0 | 100  0 | 106  2 | 98.1  1.9 |  |
| **Temporary Residency**  Yes  No | 0  15 | 0  100 | 1  72 | 1.4  98.6 | 1  19 | 5  95 | 3  105 | 2.8  97.2 |  |
| **Primary Psychology Role**  Education  Direct client services  Managerial  Not applicable  **Secondary Psychology Role**  Education  Direct client services  Managerial  Not applicable | 1  13  1  0    5  3  1  6 | 6.7  86.7  6.7  0    33.3  20  6.7  40 | 7  61  5  0    18  19  5  31 | 9.6  83.6  6.8  0  24.7  26  6.8  42.5 | 5  15  0  0  8  1  2  9 | 25  75  0  0  40  5  10  45 | 13  89  6  0    31  23  8  46 | 12  82.4  5.6  0    28.7  21.3  7.4  42.6 |  |
| **Work across sectors**  Public  Private  Both  *Missing* | 4  7  4  0 | 26.7  46.7  26.7 | 21  20  29  3 | 30  28.6  41.4 | 2  5  11  2 | 11.1  27.8  61.1 | 27  32  44  5 | 26.2  31.1  42.7 |  |
| **States of Employment**  QLD  NSW  VIC  TAS  SA  ACT  WA  NT  Multiple states  *Missing* | 3  1  5  1  0  0  2  1  2  0 | 20  6.7  33.3  6.7  0  0  13.3  6.7  13.3 | 6  20  30  2  1  1  6  2  4  1 | 8.3  27.8  41.7  2.8  1.4  1.4  8.3  2.8  5.6 | 2  0  15  0  0  2  0  0  1  0 | 10.0  0  75.0  0  0  10.0  0  0  5 | 11  21  50  3  1  3  8  3  7  1 | 10.3  19.6  46.7  2.8  0.9  2.8  7.5  2.8  6.5 |  |
| **Geographical areas of work**  Metropolitan  Regional  Rural  Remote  Multiple | 7  2  1  0  5 | 46.7  13.3  6.7  0  33.3 | 50  6  3  3  11 | 68.5  8.2  4.1  4.1  15.1 | 18  1  0  0  1 | 90  5  0  0  5 | 75  9  4  3  17 | 69.4  8.3  3.7  2.8  15.7 |  |
| **Registration or Endorsement type**  Provisional psychologist  Registered psychologist  Clinical neuropsychology  Clinical psychology  Counselling psychology  Double endorsement | 6  2  0  7  0  0 | 40  13.3  0  46.7  0  0 | 10  28  10  18  1  6 | 13.7  38.4  13.7  24.7  1.4  8.2 | 10  3  0  6  1  0 | 50  15  0  30  5  0 | 26  33  10  31  2  6 | 24.1  30.6  9.3  28.7  1.9  5.6 |  |
| **Psychology training pathway**  Doctorate degree  Master degree  5+1 Internship  4+2 Internship  Other  Missing | 3  9  0  3  0  0 | 20  60  0  20  0 | 20  31  4  15  2  1 | 27.8  43.1  5.6  20.8  2.8 | 10  7  1  1  0  0 | 52.6  36.8  5.3  5.3  0 | 33  47  5  19  2  2 | 311  44.3  4.7  17.9  1.9 |  |

**2.3 Qualitative Responses Exploring Participant Perspectives Toward Higher Education Preparedness to Work with Aboriginal and Torres Strait Islander Clients.**

| **Responses by Groups** | **Examples Quotes** |
| --- | --- |
| **No: Group 1**  **Aboriginal Psychologists** | - “Humility was not valued in the education. This is core to the work.”  - “Absolutely not. The so-called 'Cultural Awareness' 1-day workshop was traumatising as an Aboriginal person. I had my identity invalidated by 12 of the 13 other students who attended at the start of the course and the facilitators were not even Psychologists or knowledgeable about culturally competent practices within the clinical/therapeutic setting - so much that I complained to have the workshop ceased.”  - “I did my training a decade and a half ago. I receive a one hour lecture on Aboriginal people in 6 years of training. Zero training in cultural safety, zero training in culturally appropriate assessment, engagement, treatment, community care.”  - “We had one lecture on it, but it was not area specific and had few practical applications. Some elements of it were good though i.e., screening and considerations of racism etc.”  - “No, only 1–2-hour guest lectures in some subjects.” |
| **No: Group 2**  **Non-Indigenous Psychologists, with experience with Indigenous Clients** | - “A free textbook was handed to me. There were no lectures or specific guidance, definitely nothing presented by an ATSI person.”  - “I had 1 lecture on working with Aboriginal and/or Torres Strait Islander clients.”  - “I don’t recall specific training during my psychology course. I have received minimal training in my current roles and thus seek out training. A unit on culturally competent practice would have been beneficial.”  - “We had one 4-hour lecture by an Aboriginal woman, lack of follow through by universities on cultural diversity training overall.”  - “It was more tokenism than practical support or even development of cultural humility.”  - “We had cultural sensitivity training, but this did not actually teach us how to adjust clinical practice.”  - “There was some content, sparsely provided in different lectures across different subjects that often repeated the same information. However, there was some basic information about reconciliation and sewb framework etc. Some lecturers were less culturally appropriate, though some who had actually worked extensively with mob were great. None were mob though I recognise the burden it imposes if we want someone to present on their own culture. I think like many areas of psychology there wasn't enough about how to actually be with or work with Aboriginal or Torres Strait Islander clients - just some theory. When we live in a country where such inequality continues that we are on the human rights radar, we need more! And a space or place where we can ask questions.”  - “I don’t recall specific training during my psychology course. I have received minimal training in my current roles and thus seek out training. A unit on culturally competent practice would have been beneficial.”  - “Not enough. We had one unit in our masters program. It was a start, but I would have appreciated my training in case formulation and intervention from a community and social systems approach.”  - “No. Theoretical information was provided but no firsthand experiences with working in remote areas was delivered. I feel that there is a misunderstanding by academics and researchers, unless you live and work in remote places you don't understand the gravity of the situations out here.”  - “We had one 4 hour lecture by an Aboriginal woman, lack of follow through by universities on cultural diversity training overall.”  - “It was more tokenism than practical support or even development of cultural humility.”  - “We had cultural sensitivity training but this did not actually teach us how to adjust clinical practice.”  - “No, based on the training I don't feel very confident in working with this population.”  - “I do not remember any part of my training looking at aboriginal experiences.”  - “Nothing was mentioned about cultural differences in psychology.”  - “No there was nothing. I have learnt on the job and self-education. It is a major gap in our education.”  - “Graduated in 1962 but taught to think critically and examine values.”  - “We had a two-day workshop that was offensive and highly inadequate. If I took at face value what I felt they were pushing, I would have believed I should never try to support Indigenous clients because we could never comprehend them sufficiently to have even the most fundamental understanding.”  - “Not mentioned at all in my course.”  - “I felt it was to at least some extent, dependent on a particular lecturer etc, including relevant info, tended to be "one-off" sessions/lectures etc, rather than more general integration into the curriculum etc.”  - “No training in education at all regarding anything regarding working with Aboriginal and or Torres Strait Islander clients.”  - “Absolutely not. We never went near the subject.”  - “I can't recall a single lecture let alone a course or placement dedicated to this. Profoundly inadequate.”  - “I did not receive any education. I learnt from working for an Aboriginal Health organisation in the NT.”  - “We did not get any specific information.”  - “I trained 20 years ago and it was very limited in the curriculum.”  - “Issues of cultural bias and a Cultural awareness component was included but how to appropriately adjust practice and communication etc was not.”  - “In the recent Master course I completed (2017-2020), while some acknowledgement was made to be 'cultrally sensitive', no actual specific guidance was provided.”  - “Nil coursework only some brief acknowledgement.”  - “barely looked at indigenous needs unless it was a special area of interest.”  - “No a small amount of learning was dedicated to this (Lots of information with barely anything on how to put it into practise).”  - “I have certainly seen an increase from when I did my undergraduate studies to my masters studies, but I still think greater education and practice as part of our training is needed.”  - “No. We had some training - about 3 days that included trauma informed practice with Aboriginal and Torres Strait Islander peoples. However I don’t feel this was enough. I have sought more training outside of my Masters degree.”  - “Yes and no; I think that we received quite a bit of training in WA that ultimately focused on ensuring that we treat every client as an individual. I don't think there is enough resources / data available though to be able to put this into practice (e.g., lack of norms available for Aboriginal populations).”  - “I was presented with one lecture on working with people from CALD backgrounds which included some information about Aboriginal and Torres Strait Islander people. This was presented by a non-indigenous person.”  - “One or two classes within the entire program mentioned the need to consider Aboriginal and/or Torres Strait Islander clients as well as other cultural or linguistically diverse clients but with little practical guidance on how.” |
| **Yes: Group 2**  **Non-Indigenous Psychologists, with experience with Indigenous Clients** | - “Theoretically there were multiple classes across the undergraduate and postgraduate that offered useful information. However, sitting in the room with each new client is a different experience so I did not feel fully prepared in practice.”  - “We had lectures about working with this population and were always offered further training opportunities.”  - “Honest truth - most people will never actually need to know this because Aboriginal people make up 3% of Australia's population - i.e. they are not a majority of the workload.”  - “Cultural competence was a theoretical component of all subjects, providing an adequate foundation and appreciation of the need to provide culturally safe services. However, this can only be theoretical and broad. Clinicians then need to use this foundation to continually adjust their practice based on the local, specific clients they are working with.”  - “Not really. I feel like there was a lot of discussion of being aware of privilege (which is very important) but not a lot of really practical guidelines from there.” |
| **No: Group 3**  **Non-Indigenous Psychologist, with no experience with Indigenous clients** | - “Sometimes seemed like an "add on" without the chance to delve deeply.”  - “I think tertiary education is time pressured and all areas are only touched on briefly, this is the responsibility of the individual to seek further education in this area.”  - “Provided with some but would benefit from learning to help better understand/become aware of the culture/s.”  - “We were not provided with any useful information for working with Indigenous patients.”  - “I don’t feel the training was sufficient and I still don’t feel confident to work with this demographic.”  - “There was no specific training for this client group. There was some general cultural awareness training but even this was minimal and not great.”  - “Undergrad only very briefly addressed cultural competence (two lectures over four years) and the post-grad is yet to dedicate any time, although I'm sure it will before we're don't.”  - “I remember they did have some education, though there was not much practical suggestions which came from the lecture/s. I found a PD given by the APS more helpful. Though maybe that was because I was actively working with clients at that point, so I could better see how to integrate the learnings into my clinical work.”  - “There wasn’t much time spent on training to work with Aboriginal or Torres Strait Islander people. We were told about their concept of wellness and that was it.”  - “It was minimal, not even a whole subject.”  - “No education provided.”  - “There was no specific training for this client group. There was some general cultural awareness training but even this was minimal and not great.”  - “We had 1 lecture on it.” |
| **Yes: Group 3**  **Non-Indigenous Psychologist, with no experience with Indigenous clients** | - “My university education consistently incorporated working with First Nation peoples throughout all classes. We were fortunate to have a lecturer psychologist who has worked in the Kimberley with Aboriginal young people.”  - “So far, after 6 months into the masters, we have already completed a two-day cultural immersion program learning about providing services to Aboriginal and/or Torres Strait Islander clients.” |

**2.4 Additional Qualitative Responses Exploring Participants Suggestions Toward Improving Psychology Curriculum**

| **Themes (%)** | **Example Quotes** |
| --- | --- |
| **Lived Experience**  **(28%)** | - “A panel discussion involving First Nations people with a lived experience of social and emotional wellbeing concerns discussing their experiences engaging with services” (Participant 9, Group 3).  - “lived experience perspectives.” (Participant 8, Group 3).  - “Asking a Subject Matter Expert. Seeking another mental health professional (preferably one of Aboriginal or Torres Straight Island background) that I can seek support, ask questions to improve my practice.” (Participant 12, Group 3).  - “More opportunities to hear from Aboriginal and/or Torres Strait Islander people about what they have found valuable and problematic in their mental healthcare experiences.” (Participant 28. Group 3).  - “Speaking with lived experience clients would be very helpful to know what they found useful or what they disliked.” (Participant 63, Group 3). |
| **Education**  **(24%)** | “I think it is important for everyone to have a better understanding of the specific impacts of the intergenerational trauma experienced by this population; I think most psych’s are aware this is a 'thing', though don't actually understand what this looks like for the individual (e.g., fear and mistrust of accessing health care even if it is available, because of the risks faced when accessing these services in the past)” (Participant 11, Group 3)  - “When it's necessary to seek supervision from a psychologist who is experienced working with Aboriginal and/or Torres Strait Islander clients in order to ensure you are working within your competencies and working most effectively for that client. Also, I think it is important for everyone to have a better understanding of the specific impacts of the intergenerational trauma experienced by this population; I think most psych’s are aware this is a 'thing', though don't actually understand what this looks like for the individual (e.g., fear and mistrust of accessing health care even if it is available, because of the risks faced when accessing these services in the past).” (Participant 14, Group 3)  - “Learn more about the culture/s and how cultural norms and experiences may influence help sealing and therapeutic relationships/approaches.” (Participant 33, Group 3).  - “What community structures can look like and the supports within the community.” (Participant 38, Group 3).  - “Decolonisation training. Building awareness of implicit bias. Training in appropriate communication, measures & therapeutic approaches.” (Participant 70, Group 3).  - “Useful language to use, but mostly the important foundations of cultural factors to consider when providing psychological services to Aboriginal and/or Torres Strait Islander clients.” (Participant 76, Group 3).  - “general knowledge, not stereotyping (not everyone feels connected to Country) -e.g., how to work with young people vs older clients; clients living rural areas vs clients living in metropolitan areas; protocols.” (Participant 92, Group 3). |
| **Practical Exercises (20%)** | - “Building awareness of implicit bias. Training in appropriate communication, measures & therapeutic approaches” (Participant 65, Group 3)  - “I would appreciate opportunities to consider how to apply models like the SEWB in practice and prompts that I might use to deepen my self-reflection to more thoroughly examine how my way of working may be able to be tailored to better support Aboriginal and/or Torres Strait Islander clients.” (Participant 28. Group 3) |
| **Intervention Guidelines (28%)** | - “Guidelines/recommendations about potentially useful adaptations” (Participant 33, Group 3).  - “Whether or not approaches like CBT or EMDR are culturally appropriate. Or which modalities might be most culturally appropriate.” (Participant 44, Group 3).  - “I've been to a few workshops on working with this group and it seems like theres a lot that needs to be adapted in practice. Perhaps a bit of guide on where to start and different levels of competency that we can aim to achieve with this group.” (Participant 87, Group 3). |

**2.5 Additional Qualitative Responses Exploring Non-Indigenous Participants Responses Toward Develop their Cultural Knowledge**

| **Main Themes** | **Example Quotes** |
| --- | --- |
| **Supervision** | - “Continuing to seek supervision from ALOs” (Group 2)  - “Continual supervision of practice” (Group 2)  - “Peer supervision with Aboriginal and Torres Strait Islander peers” (Group 3) |
| **Workshops or conferences** | - “Will look into further professional development if/when I get a referral for indigenous client” (Group 3)  - “Additional training in culturally appropriate assessments” (Group 3)  - “Engage with online cultural training courses” (Group 2)  - “I attended Lowitja conference” (Group 2)  - “Always looking out for affordable training in this area.” (Group 2)  - “I've been doing some online training in Narrative Therapy recently. And there are often different research presentations about communication around so I go to those when they come up.” (Group 2) |
| **Learning from Indigenous peers or clients** | - “Continue to listen to my Aboriginal and/or Torres Strait Islander clients and coworkers” (Group 2)  - “In every encounter I have to learn something new” (Group 2)  - “Being open to interaction in my "other" life.” (Group 3)  - “I seek out supervision from a local Indigenous psychologist” (Group 3)  - “Informally via continuing to work with indigenous populations” (Group 2)  - “I have an intern who works in this area and am learning from her” (Group 2)  - “Ongoing discussions and consultation with Aboriginal peers and cultural liaison workers” (Group 2)  - “Continuing to work in same remote communities for foreseeable future” (Group 3)  - “I want to attend a workshop by an Aboriginal or Torres Strait Client” (Group 3)  - “Through further remote work and learning from communities” (Group 2)  - “More experience of working with and learning from this client group” (Group 3)  - “Ongoing PD and peer supervision with Aboriginal &Torres Strait Islander peers.” (Group 2)  - “Through further remote work and learning from communities. I am also interested in growing the evidence base by completing my own research in the future.” (Group 2)  - “Culture mentorship.” (Group 2)  - “Yes - I want to attend a workshop by an Aboriginal or Torres Strait Client.” (Group 2) |
| **Participate in Indigenous focused activities** | - “Ongoing involvement in Aboriginal cultural activities in my local area and in conjunction with my service” (Group 2)  - “Engagement in cultural events at work such as Reconciliation week” (Group 2)  - “Continue to participate in the Reconciliation Action Plan Working Group” (Group 3)  - “No plan yet for how but hopefully some way to connect with actual mob on country.” (Group 2) |
| **Exploring research and grey literature** | - “At this stage in my career my knowledge shift is coming more from reading research undertaken by Aboriginal Psychologists about Aboriginal consumers” (Group 2)  - “Research of specific ideas” (Group 3)  - “Academic research” (Group 2)  - “By continuing to read relevant books, biographies, and movies” (Group 2) |
| **Participants that responded ‘no’ or had no direct plans to increase their knowledge** | - “I prefer to use my professional development time upskilling in things that can help ALL of my patients - not focusing on thangs that make a small difference to a very small minority.” (Group 2)  - “Don't overcomplicate things. If you ivory tower researchers want to make a buck for yourself you can write a thesis, but treating people like people is a basic skill.” (Group 2)  - “Hope I do but will not pursue that at the expense of working appropriately with whoever walks through my door.” (Group 2)  - “probably incidentally, not by attending any more workshops, etc, at this point as I already have enough to work on with my core business.” (Group 2)  - “I have completed some training on this as part of general orientation when starting my job as a psychologist. I do not plan to complete further professional development in this area unless I start working with such clients.”  - “I think I have enough.” (Group 2)  - “No, I have done substantial training already.” (Group 2) |

**2.6 Additional Qualitative Responses Exploring Non-Indigenous Cultural Questions**

| **Aligning with Paper** | **Example Quotes** |
| --- | --- |
| **Easily Addressed** | “What language/terminology is preferred, and are there aspects of standard psychological practices that might be ill-fitting or mismatched to culture??” (Participant 8, Group 3).  “How do these preferences differ between First Nations populations, noting that, for example, the Wurundjeri people are different from the Ngunnawal people.” (Participant 9, Group 3).  “How do I treat an ATSI client differently from all clients? I am unsure if I need to do different things to be culturally aware when I feel I already do so for every client and don't discriminate positively or negatively based on culture.” (Participant 48, Group 2). |
| **Specific questions about Indigenous psychology** | “I think I have good training and understanding in history, experiences of Aboriginal and/or Torres Strait Islander clients, etc etc but very little training in practically, how to adapt practice to be more culturally safe (e.g., what do I actually do in the session).” (Participant 23 in Group 2)  “How mainstream psychotherapeutic practices are best adapted to assist Aboriginal clients. How this can be taught. How such resources can be built.” (Participant 60 in Group 2).  “How best to use/adapt psychological knowledge to best support them.” (Participant 37 in Group 3)  “I would very much like a framework for neuropsychology to work with Aboriginal and Torres Strait Islander peoples.” (Participants 74, in Group 2).  “Culturally appropriate neuropsychology assessment.” (Participants 81 in Group 2).  “More information on the validity and utility of neuropsychology tests in this population would be helpful.” (Participant 108 in Group 2) |
| **Culturally specific questions** | “I would like to know more about cultural practices like men’s business and women’s business, etc. It’s like the practices are named but not explained.” (Participant 10 in Group 2)  “More about things that actually show up in the room, like family structures and gender roles and ways to build or explore identity as an Aboriginal or Torres Strait Islander person. Ways of healing, ways to approach elders, culturally normative experiences that could be mistaken for mental ill-health.” (Participant 25 in Group 2).  “I think I would like to be told clearly "this is how you ask someone about their dreaming/totem, cultural healing practices, etc.” (Participant 45 in Group 2). |
| **Existential questions** | “How can a 'white person' ever hope to be able to provide meaningful therapy when there is such a divide. It feels like appropriation to use words like 'mob' or try 'yarn'. We are taught these words and even suggested that we try yarning, but how do we do that in a way that is respectful rather than inappropriate?” (Participant 11 in Group 2).  “When will universities stop pretending that Western psychology, and particularly psychiatry is, without significant change, appropriate for Aboriginal people?” (Participant 56 in Group 2). |
| **Slightly outside of the scope** | “How to combat racism from other clients?” (Participant 62 in Group 2).  “How to show I'm different? I'd like to be able to make it clear that I'm a supporter and an ally, but I worry about touting myself because I know so many people say one thing but do another, and so many of my client’s experience that before they get to me.” (Participant 73 in Group 2).  “Why perpetrators of (Domestic and Family Violence) DFV are often protected by the community and the survivor is often further abused for trying to leave.” (Participant 59 in Group 2).  “Is your identification as an Aboriginal and/or Torres Strait Islander person part of the reason for your decision to seek psychological support? This would allow me to triage whether I am competent to work with them as I currently work, or whether I work with them and get an external supervisor more experienced in this area, or whether I refer them on to someone better suited” (Participant 14 in Group 3).  “Many, but none specifically. This feels like a role that requires a large time commitment and could not be a transient role.” (Participant 63 in Group 3). |

**3. Key Recommendations**

1. Australian psychology higher education training desperately needs to develop a plan to better prepare non-Indigenous psychology students to work with Aboriginal and Torres Strait Islander peoples. This should be addressed through a number of means including:
   1. Employ Aboriginal and Torres Strait Islander peoples to sit within the school of psychology to support Indigenous psychology students and guide educators’ learnings when possible.
   2. Psychology educators cannot teach what they do not know; develop learning opportunities to enhance educators’ cultural responsiveness through,
      1. Fostering nourishing relationships with the local Aboriginal communities to enhance adequately reimbursed learning opportunities.
      2. Developing a dynamic learning process with psychology educators to minimise resistance behaviours.
      3. Providing opportunities for educators to develop critical self-reflection skills for greater insights into the biases that they hold and inadvertently transfer to students.
      4. Being driven by students’ desire to learn about Aboriginal and Torres Strait Islander mental health and wellbeing and be prepared to meet that desire.
      5. Accessing the educational resources at your disposal (e.g. AIPEP and IAHA resources)
   3. Psychology educators need to use these learnings to redevelop the curriculum considering the following aspects.
      1. Consider incorporating Indigenous knowledges, theories, frameworks, perspectives and methods.
      2. Removing deficit discourses, generalisations, and stereotyping of Aboriginal and Torres Strait Islander peoples
      3. Ensuring the development of student’s critical self-reflection tools
      4. Develop students' critical thinking skills to the application of psychology ethical guidelines and interventions
      5. Introduce the universities terminology resources in the early stages of student’s university careers
      6. Incorporating Aboriginal and Torres Strait Islander peoples lived experiences and stories
      7. Ensuring the processors of colonisation and Australian history, as well as the continuing impacts and outcomes this has had on Aboriginal and Torres Strait Islander peoples is a core element of first year psychology subjects.
      8. Privileging Indigenous voices, expanding beyond a guest lecture by an Indigenous person and purely academic literature. Preferencing a continual process that can be facilitated through the recommended readings provided (e.g. books, poems, artwork, and songs).
      9. Incorporate more practical exercises, for students to actively engage with Aboriginal and Torres Strait Islander content and frameworks.
      10. Take a strengths-based approach when working with and teaching about Aboriginal peoples by framing our culture as a protective factors and privileging healing over trauma.
      11. Educating about the diversity within the Aboriginal populations is necessary to understand there are no universal guidelines or instructions manuals that will be sufficient to work with all Aboriginal and Torres Strait Islander clients. Learning to work reflexively is key.
      12. Reframing the impact of a client’s race to the impact of racism on clients to normalise it as a human experience, in turn working to reduce the apprehension students hold about working with Aboriginal and Torres Strait Islander clients.
      13. Ensure students know how to find appropriate mental health assessments for all clients, and the process to engage with in the absence of a valid and reliable measure.
   4. Schools of Psychology, and educators working within it may find it useful to develop their own accountability measures. This ensures progress towards anti-racist higher education psychology student experiences.
2. Current psychology students and graduates’ psychologists need to recognise the gaps in their higher education training, and address these gaps to become culturally responsive, and reflexive psychologists.
